# The importance of wellness among users of complementary and alternative medicine: findings from the 2007 National Health Interview Survey

**DOI:** 10.1186/s12906-015-0886-y

**Published:** 2015-10-15

**Authors:** Dawn M. Upchurch, Bethany Wexler Rainisch

**Affiliations:** Department of Community Health Sciences, UCLA Fielding School of Public Health, 650 Charles Young Drive South, Los Angeles, CA 90095-1772 USA; Department of Health Sciences, California State University, 18111 Nordoff Street, Northridge, CA 91330 USA

**Keywords:** CAM, Wellness, Health promotion, Sociobehavioral wellness model, Lifestyle, Health care

## Abstract

**Background:**

This study developed and tested a sociobehavioral wellness model of complementary and alternative medicine (CAM) to differentiate predisposing factors, enabling resources, need, and personal health practices according to use for wellness, for combined wellness and treatment, or for treatment alone.

**Methods:**

Data were from the 2007 National Health Interview Survey (NHIS), a cross-sectional, nationally representative sample of 23,393 adult Americans. This analysis included people who used at least one CAM modality in the past 12 months (*n* = 7003 adult users). Prevalence estimates and multinomial logistic regression results were weighted and adjusted for complex sample design.

**Results:**

Overall, 86 % of CAM users reported reason for use as wellness (51 %) or wellness combined with treatment (35 %). White women had the lowest (48 %) and Asian men (66 %) had the highest wellness use. Compared to treatment only users, wellness users were significantly more likely to be older, more educated, in better health, and engaged in multiple healthy behaviors. There was support that those with health conditions were using methods for both treatment and to maintain health.

**Conclusions:**

The findings underscore the central role of CAM in health self-management and wellness lifestyle. At a time of national health care reform highlighting the importance of health and wellness and employers turning to wellness programs to improve worker performance and well-being, these findings suggest a central role of CAM in those public health endeavors.

## Background

Use of CAM is increasingly part of Americans’ health care and health-seeking practices. Use is common and has significantly increased over the past decade, with close to 40 % in 2007 using some form of CAM in the past year [1–3]. CAM encompasses a range of products, practices, and providers [4], almost all of which have a wellness or health promotion component. Accordingly, individuals’ reasons and motivations for use are varied [5–7]. Some may use CAM for wellness and as part of a healthy lifestyle while others with health conditions may use it for treatment of those conditions [5, 7, 8]. Historically, researchers have framed reasons for use in the context of a problem-based, treatment focus. More recently, there is increasing recognition for the need to more fully elaborate the diversity of motivations and reasons for use. In particular, framing CAM use as health self-management and as a part of a wellness lifestyle is a useful approach [4, 6, 9–12]. Better understanding and characterizing use for wellness has considerable public health significance with respect to health promotion and disease prevention in light of the burden of lifestyle diseases in the United States.

Informed by earlier work [4, 12–16] we apply a sociobehavioral wellness model of CAM use to comprehensively evaluate the differences in reasons for use. Specifically, we ask: Can the sociobehavioral wellness model be used to further differentiate predisposing factors, enabling resources, need, and personal health practices among CAM users with regard to their reason for use? We distinguish the characteristics of those who use CAM for wellness, for both wellness and treatment combined, or for treatment only.

### Sociobehavioral wellness model of CAM use

Variations of sociobehavioral models of CAM use, including our own, have been described and some empirically tested [4, 13–18]. These models are applications and extensions of the well-known Andersen Behavioral Model of health services utilization of conventional care [19–21]. Here, we briefly review the components of our model. Use of CAM is influenced by four distinct domains. Predisposing factors include demographic characteristics as well as knowledge, attitudes, and beliefs about CAM and about health and illness. Enabling resources reflect the ability to obtain and use health services, and include factors such as income, health insurance, and accessibility to conventional (and alternative) health services. Need includes subjective and objective assessment of medical need. Finally, personal health practices consist of individuals’ overall lifestyle and health practices and reflect behavioral manifestations of health beliefs. Our sociobehavioral wellness model of CAM proposes that predisposing factors are exogenous, enabling resources are necessary but not sufficient conditions for use, that some need (including desire for wellness and health promotion) must be defined for use to occur, and that personal health practices are influenced by factors in each of the previous domains. We also propose that these domains will inform and differentiate CAM users with respect to use for wellness, for wellness and treatment, or for treatment alone.

Previous research shows that many of the characteristics associated with the use of conventional care are also associated with use of CAM [4, 10, 12, 17, 18, 22–26], thus demonstrating the usefulness of a unified conceptual approach to understanding a multiplicity of health-care seeking behaviors. In general, women, whites, those with higher socioeconomic status, with greater health needs, better access to conventional care, and those with healthier lifestyles are more likely to use CAM [4, 10, 12, 17, 18, 23–27]. Among users, a few studies have considered reasons for use, especially in the context of wellness [10, 12, 17, 18]. The current study extends the previous research by investigating the effects of a multiple characteristics within each of the four domains of the sociobehavioral wellness model to provide a comprehensive picture of users for wellness, both wellness and treatment, or for treatment alone. Because the effects of gender on reason for use may depend on race/ethnicity, we also examine gender-by-race/ethnicity interactions.

### CAM Use as health self-management

Our sociobehavioral wellness model also considers CAM as one aspect of healthy self-care [6, 8–13]. Health self-management refers to activities individuals engage in to maintain health and wellness, to prevent illness and disease, or to manage illness and disease. Differentiating CAM use for wellness and health versus treatment is essential because factors that contribute to each type of use are likely to differ.

We view health as a biopsychosocial phenomenon, encompassing social, psychosocial, and physical components [28], not simply the absence of disease. Rather than conceptualize health as a neutral condition that is recognized only under conditions of threat (i.e., illness), we identify positive health as a dynamic asset with the potential capacity to resist illness [29]. Wellness has been described as a higher order construct [6]. Definitions vary, but there appears to be a growing consensus that wellness is a positive approach to living that is multidimensional, holistic, and consciously self-directed [30]. These ideas are in line with core tenets of many types of CAM that focus on balance and harmony in all aspects of one’s life, thus allowing the body to cope with life stressors and heal [6]. Many types also explicitly promote self-care, self-monitoring, and empowerment. Thus, some users may engage in healthy lifestyle practices not just for disease prevention but because improved wellness is viewed as an end in itself [5–7]. Thus, the notion of “wellness lifestyles” [6] that links healthy lifestyles and the use of CAM for wellness is an invaluable component of our sociobehavioral wellness model of CAM. In this study we are especially interested in the extent to which perceived and evaluated health and the degree to which individuals engage in multiple healthy lifestyle behaviors contribute to their use for wellness.

For the current study, we hypothesize that the effects of gender are contingent upon race/ethnicity and based on prior research, we propose white women would have the highest rates of use for wellness. We anticipate that older users would be more likely to report use for combined wellness and treatment, and less so for wellness alone. Also, those with higher socioeconomic status will be more likely to be wellness users. Last, we hypothesize that users with poorer health would be less inclined to be wellness users, while those who engage in healthy lifestyle behaviors more likely.

## Methods

### National Health Interview Survey (NHIS), 2007

The NHIS is an annual, cross-sectional, in-person household interview survey of US civilian, non-institutionalized population [31]. The survey uses a multi-stage clustered sample design with oversamples of blacks, Asians, and Hispanics. Basic health information is collected on one randomly selected adult age 18 or over (Sample Adult Core) in each household. The adult response rate for the 2007 sample was 67.8 % [32]. The 2007 survey included a CAM supplement administered to the Sample Adult [2, 32]. Individuals in the Sample Adult Core (*n* = 23,393) were questioned about 36 types of specific CAM modalities [2]. For each of these 36 modalities, individuals were asked if they had ever used it in their lives, and if yes, if they had used it in the past 12 months. A list of the specific CAM modalities is provided in the Appendix. Individuals who reported race or ethnicity as “other” (*n* = 244) were excluded because of small sample size and heterogeneity (*n* = 23,149). The final analytic sample includes people who used at least one CAM modality in the past 12 months, and for whom there were valid data on reason for use (*n* = 7003). This research was approved by UCLA Office of the Human Research Protection Program.

### Measures

#### CAM use measures

The use of any type of CAM in the past 12 months was defined as “recent use” and is what is analyzed here. Recent users were asked more detailed information regarding the nature and reasons for use. Specifically, they were asked if they use CAM for treatment of a specific health condition. They were also asked if they used CAM “to improve or enhance energy,” “for general wellness or general disease prevention,” or “to improve immune function.” Mention of any one of these three reasons was coded as “wellness.” If a given user only mentioned wellness for all methods used, they were coded as “wellness only.” If a given user also mentioned use for treatment for some of the methods they used, they were coded as “both wellness and treatment.” If a given user only mentioned treatment for all methods used, they were coded as “treatment only.” The outcome measure was an assessment of reason for use and was coded as: wellness only (*n* = 3572), both treatment and wellness (*n* = 2458), and for treatment only (*n* = 973). It was coded as a trichotomous variable.

#### Predisposing factors

Demographics included gender, race/ethnicity (white, black, Hispanic, Asian), nativity status (US born or not), age (10-year age categories except for the youngest and oldest categories), education (less than high school, high school, more than high school), and marital status (never married, married, cohabiting, divorced/widowed). We also created an 8 category gender-by-race/ethnicity interaction variable.

#### Enabling resources

These included household income (≤$34,999, $35,000–49,999, $50,000–74,999, $75,000–99,999, ≥$100,000), current health insurance status (public, private, uninsured), whether conventional care was delayed or not received because of cost (yes, no), whether there was a usual place of care (yes, no), and US Census Bureau geographic region (Northwest, Midwest, South, West).

#### Need

Subjective health was based on self-reported health status (excellent, very good, good, fair, poor), and objective health status based on number of diagnosed chronic health conditions (0, 1–2, 3–5, 6+).

#### Personal health practices

Four variables were considered and standard coding was used. Leisure time physical activity (regular activity: light or moderate activity performed at least 30 min 5 or more times per week and/or vigorous activity performed at least 20 min 3 or more times per week; some activity: less than regular, but more than none; and no activity). Smoking status (current: smokes every day or some days; former: smoked at least 100 cigarettes in life but not in past 30 days; never: smoked less than 100 cigarettes in lifetime). Alcohol consumption status (lifetime abstainer: ≤12 drinks in lifetime; former: 0 drinks in past year, but ≥12 in lifetime; current infrequent/light: ≤ 3 drinks per week; current moderate/heavy: > 3 drinks per week). Body mass index (BMI) (underweight: <18.5; healthy: 18.5 to 24.9; overweight: 25.0 to 29.9; obese: >30). A healthy behavior index was constructed and was a summary measure of these four variables. First, each of the four variables were dichotomized, with 1 representing the “healthiest” category for each (some or regular leisure time physical activity, never or former smoker, abstainer, former or current light drinker, and healthy BMI). Then, the four dichotomous variables were summed and the final measured varied from 0 to 4.

#### Analysis

All analyses and estimates used the individual-level sampling weights that adjust for nonresponse and poststratification. Variance estimates were adjusted to account for complex sample design [33]. Descriptive statistics and bivariate prevalence estimates were estimated using the design-based F test. Weighted multinomial logistic regression was used to estimate the effects of predisposing factors, enabling resources, need, and personal health practices on use for treatment, wellness, or for both treatment or wellness. In this analysis, use for treatment is the referent category. For ease of presentation, adjusted odds ratios (AORs) are presented. All analyses were performed using Stata 12.0 [33].

## Results

### Descriptive results: distributions of population and CAM users

Close to 40 % reported using CAM in the past 12 months during 2007 (Table 1). Column 1 shows the weighted distribution of the full sample of adults Column 2 shows the weighted distribution of recent users. Except for marital status, there were significant differences between the two groups for all other variables in each of the four model domains. Higher percentages of white women, native born, older, and those with higher education and income were recent users. Those with somewhat greater access to conventional care also had higher percentages of use. Higher percentages of those reporting better perceived or lower evaluated health were recent users. Last, those practicing greater number of healthy behaviors had higher percentages of recent use.

**Table 1:** Distributions of full sample and recent CAM users, NHIS, 2007

	Sample % (*n* = 23,149)	Recent users (*n* = 7003)
Total	100.0	38.9
Predisposing Factors		
Gender-by-Race ***		
White Females	39.3	49.4
Black Females	7.5	5.0
Hispanic Females	5.8	3.7
Asian Females	1.9	2.4
White Males	33.4	31.9
Black Males	5.0	3.0
Hispanic Males	5.3	2.9
Asian Males	1.8	1.7
Nativity Status ***		
United States born	86.1	90.7
Foreign-born	13.9	10.3
Age ***		
18–29	19.7	17.5
30–39	17.6	17.9
40–49	18.7	19.6
50–59	16.5	20.1
60–69	13.0	13.7
70+	14.5	11.3
Education ***		
< 12 years	15.0	7.8
High school graduate	29.1	21.8
> 12 years	55.9	70.4
Marital Status		
Never married	22.5	22.0
Married	46.5	47.1
Cohabiting	5.3	5.1
Divorced/Separated/Widow	25.7	25.8
Enabling Resources		
Income ***		
0–$34,999	46.0	38.0
$35,000–$49,999	13.6	13.2
$50,000–$74,999	17.5	19.5
$75,000–$99,999	9.3	10.9
+ $100,000	13.6	18.4
Insurance Status ***		
Private	66.4	72.8
Public	18.3	14.7
Uninsured	15.3	12.5
Delayed care because could not afford ***		
Yes	11.6	15.1
No	88.4	84.9
Did not receive care because could not afford ***		
Yes	8.7	10.7
No	91.3	89.3
Usual place for health care ***		
Yes	85.6	87.4
No	14.4	12.6
Region ***		
Northeast	17.3	17.3
Midwest	25.2	26.3
South	36.7	30.4
West	20.8	26.0
Need		
Perceived Health Status ***		
Excellent	27.9	29.0
Very Good	32.0	34.3
Good	26.1	23.7
Fair	10.4	10.0
Poor	3.6	3.0
Health Conditions ***		
0	36.3	27.7
1–2	34.2	37.1
3–5	20.4	24.0
6+	9.1	11.2
Personal Health Practices		
Healthy Behavior Index ***		
0	1.3	0.9
1	9.9	8.3
2	35.1	29.1
3	39.6	43.7
4	14.1	18.0
Continuous Healthy Behavior Index*** Mean (SD)	2.55 (.01)	2.70 (.01)

Percentages are weighted to US population estimates. Design-based F test for bivariate analysis

***p ≤ .001

### Comparison of reasons for Use: bivariate results

Over half of recent users reported using CAM for wellness only (Table 2). Another third reported using CAM for both wellness and treatment of a specific health condition. Only 14 % of people used CAM for treatment only. At the bivariate level, all variables in each of the four domains were significantly associated with reason for use. Of all gender-by-race groups, white women had the lowest percentage of use for wellness alone; Asian men had the highest percentage, followed closely by Asian women and black men. White women had the highest percentage of combined users. A higher percentage of foreign-born reported use for wellness and combined use was more common among native born. Almost two-thirds of the youngest adult users reported wellness as reason for use and the percentages were lower among older adults. The percentages reporting combined use was higher among older adults. Use for wellness was higher for both higher levels of education and income. For marital status, the highest percentage of wellness use was among never-married individuals. Individuals with greater access to conventional care reported highest percentages of use for wellness; there was little real difference in percentages of use for wellness by region. Wellness use was lower for lower perceived health status declined and for greater number of health conditions. Last, mean number of health behaviors was higher among wellness and combined users.

**Table 2:** Prevalence of use for wellness, for both wellness and treatment, or treatment only among CAM users NHIS, 2007 (*n* = 7003)

	Wellness %	Both wellness and treatment %	Treatment only %
Total	51.0	35.1	13.9
Predisposing Factors			
Gender-by-Race ***			
White Female	48.4	38.5	13.1
Black Female	60.3	26.8	12.9
Hispanic Female	52.2	34.3	13.5
Asian Female	65.8	28.0	6.2
White Male	50.5	33.2	16.3
Black Male	63.1	27.7	9.2
Hispanic Male	50.5	30.9	18.6
Asian Male	66.2	27.9	5.9
Nativity Status ***			
United States born	50.2	35.8	14.0
Foreign-born	58.3	29.4	12.3
Age ***			
18–29	62.8	22.8	14.4
30–39	55.4	31.3	13.3
40–49	46.9	39.6	13.5
50–59	48.6	38.5	13.0
60–69	45.2	40.7	14.1
70+	44.2	39.7	16.1
Education ***			
< 12 years	38.6	38.2	23.1
High school graduate	45.9	36.6	17.4
> 12 years	54.0	34.3	11.3
Marital Status***			
Never married	59.6	28.7	11.7
Married	50.6	34.3	15.1
Cohabiting	52.3	35.2	12.5
Divorced/Separated/Widow	44.1	42.1	13.9
Enabling Resources			
Income **			
0–$34,999	48.7	36.4	15.0
$35,000–$49,999	46.4	38.4	15.2
$50,000–$74,999	52.0	34.7	13.3
$75,000–$99,999	55.1	32.4	12.5
+ $100,000	55.6	32.3	12.2
Insurance Status ***			
Private	53.2	33.2	13.6
Public	40.0	44.6	15.5
Uninsured	51.4	34.9	13.8
Delayed care because could not afford ***			
Yes	41.9	43.1	15.1
No	52.6	33.7	13.7
Did not receive care because could not afford ***			
Yes	40.1	46.3	13.9
No	52.3	33.8	13.6
Usual place for health care ***			
Yes	49.8	36.1	14.1
No	59.3	28.6	12.1
Region ***			
Northeast	52.3	34.0	13.7
Midwest	48.7	34.3	17.1
South	53.7	32.5	13.8
West	49.4	39.8	10.9
Need			
Perceived Health Status ***			
Excellent	63.2	26.6	10.2
Very Good	54.1	32.7	13.4
Good	43.8	39.5	16.7
Fair	31.5	50.4	18.2
Poor	20.4	59.0	20.6
Health Conditions ***			
0	65.8	22.9	11.3
1–2	52.4	33.8	13.8
3–5	41.6	43.5	14.9
6+	30.0	51.6	18.4
Personal Health Practices			
Healthy Behavior Index ***			
0	45.4	28.2	26.4
1	42.4	37.7	19.8
2	47.4	36.0	16.6
3	51.5	36.4	12.1
4	59.2	30.0	10.8
Continuous Healthy Behavior Index ***	2.8 (0.02)	2.7 (0.02)	2.5 (0.03)
Mean (SD)			

Percentages are weighted to US population estimates. Design-based F test for bivariate analysis

***p ≤ .001

### Comparisons of reasons for use: multivariate results

#### Predisposing factors

Compared to white women, Asian women and men and black men were more likely to report wellness use versus treatment alone. Compared to white women, white and Hispanic men were less likely to report combined use. Nativity status was not significant. When compared to the youngest age group, older ages, especially middle age and older were more likely to use CAM for wellness and for combined wellness and treatment rather than treatment alone. This was especially true for older individuals using CAM for combined wellness and treatment. Individuals with the highest education were more likely than individuals with the least education to use CAM for wellness or combined wellness and treatment versus treatment alone. Never married persons were more likely than married to report use for wellness and combined wellness and treatment than treatment alone. Never-married, divorced, and cohabiting individuals were more likely than married to report use for both wellness and treatment.

#### Enabling resources

At the multivariate level, the majority of the enabling resources variables were not significantly associated with reason for use. However, compared to treatment alone, users who said they did not receive conventional care because of cost were more likely to use for combined wellness and treatment. Those living in the west were more likely to use for wellness and treatment combined versus treatment alone.

#### Need

Compared with persons reporting excellent health, those reporting good, fair, or poor were less likely to use CAM for wellness versus treatment alone. There was no health status difference between those reporting use for both wellness and treatment compared to treatment only. Similarly, compared to persons reporting no chronic health conditions those reporting health conditions were less likely to use CAM for wellness versus treatment alone. However, those with the more health conditions were somewhat more likely than those with none to report use for both wellness and treatment combined.

#### Personal health practices

Last, increasing numbers of healthy behaviors was significantly associated with a greater likelihood of using CAM for wellness alone, or for wellness in combination with treatment, versus treatment alone (Table 3).

**Table 3:** Adjusted odds ratios of multivariate analyses comparing characteristics according to reason for use, NHIS, 2007 (*n* = 7003)

	Wellness only versus treatment only	Both wellness and treatment versus treatment only
Predisposing Factors		
Gender-by-Race		
White Females	1.00	1.00
Black Females	1.38	0.72
Hispanic Females	1.15	0.86
Asian Females	2.20**	1.46
White Males	0.87	0.74***
Black Males	2.07**	1.09
Hispanic Males	0.79	0.64*
Asian Males	2.28*	1.57
Nativity Status		
United States born	1.00	1.00
Foreign-born	1.04	0.87
Age		
18–29	1.00	1.00
30–39	1.22	1.73***
40–49	1.21	2.16***
50–59	1.50**	2.10***
60–69	1.64**	2.17***
70+	1.55*	1.81**
Education		
< 12 years	1.00	1.00
High school graduate	1.39*	1.30
> 12 years	1.92***	1.80***
Marital Status		
Never married	1.60**	1.49**
Married	1.00	1.00
Cohabiting	1.41	1.54*
Divorced/Separated/Widow	1.16	1.29*
Enabling Resources		
Income		
0–$34,999	1.00	1.00
$35,000–$49,999	0.85	1.09
$50,000–$74,999	1.03	1.13
$75,000–$99,999	1.18	1.14
+ $100,000	1.10	1.12
Insurance Status		
Private	0.91	0.83
Public	0.87	1.03
Uninsured	1.00	1.00
Delayed care because could not afford		
Yes	0.74	0.86
No	1.00	1.00
Did not receive care because could not afford		
Yes	1.18	1.56*
No	1.00	1.00
Usual place for health care		
Yes	1.00	1.00
No	1.29	1.06
Region		
Northeast	1.00	1.00
Midwest	0.82	0.83
South	1.12	0.96
West	1.17	1.42*
Need		
Perceived Health Status		
Excellent	1.00	1.00
Very Good	0.76*	0.93
Good	0.56***	0.90
Fair	0.45***	1.04
Poor	0.29***	1.02
Health Conditions		
0	1.00	1.00
1–2	0.76*	1.19
3–5	0.67**	1.41*
6+	0.56***	1.38
Personal Health Practices		
Continuous Healthy Behavior Index	1.20***	1.20***

Weighted multinomial regression

*p ≤ .05; **p ≤ .01; ***p ≤ .001

## Discussion

This is one of the first studies to comprehensively distinguish CAM users based on their reason for use, with an emphasis on wellness. A substantial majority (86 %) reported use for wellness or wellness and treatment combined and over half of reported wellness alone as their reason for use. The multivariate analysis shows significant associations of multiple predisposing factors, need, and personal health practices in differentiating users for wellness, for wellness and treatment, or for treatment alone. As a domain, the variables operationalizing enabling resources, do not significantly distinguish types of users. With regard to personal health practices, those engaging in a greater number of healthy behaviors are more likely to use CAM for wellness or combined wellness and treatment. Taken together, these findings demonstrate the potential usefulness of the sociobehavioral wellness model for CAM use and unequivocally point to the need to frame CAM use as health self-management and as part of a wellness lifestyle. Importantly, there is a critical need to continue to develop and empirically test measures that characterize the multidimensionality of the concept of “wellness” [30].

### Predisposing factors

The effects of gender are contingent upon race and surprisingly, white women report the lowest percentage of wellness users while black and Asian men report some of the highest percentages. In fact, black men are over twice as likely as white women to be wellness users. Asian men and women are also more likely to be wellness users. And among combined users, only white and Hispanic men are less likely than white women. Few studies have examined gender-by-race interactions with regard to CAM use, let alone reasons for use. There is clearly non-random selection by gender and race with respect to use [2, 22, 34] as well as unobserved differences among users and non-users. One study, using the 2002 NHIS and analyzing only African Americans, found that users of CAM were more likely to be older, female, better educated, insured, and have higher income [35]. However, they also found that the “majority” used CAM for treatment rather than health promotion, although they did not provide statistical analysis for this conclusion. Importantly, the 2002 NHIS did not explicitly ask specific questions pertaining to health and wellness, only about treatment, thus potentially underestimating use for wellness. Also, it does not appear that black users are more likely than white users to utilize modalities that might be thought of as more “wellness focused” (e.g., relaxation techniques, yoga, supplements) [23]. Prayer for health was not included as a CAM modality in the 2007 NIHIS, and other research suggests it is commonly used among black men and women. The findings for Asian men and women are less surprising given the focus on prevention and wellness in Traditional East Asian Medicine and the commonality of practices such as tai chi and meditation in this population [36].

Unfortunately, the NHIS does not include information on specific motivations and beliefs known to be associated with use which may be useful in understanding these gender and racial/ethnic differences [5, 7, 37]. An early, high-quality national survey found that users of CAM were more likely to have unconventional religious beliefs and be in a category the author called “cultural creative” [37]. Users also had greater desire for control over their health. In a more recent systematic review of beliefs among CAM users [5], the authors summarized both quantitative and qualitative studies. In general, users viewed CAM as part of self-management for those with chronic health conditions, believed psychological factors had a role in the origins of illness and the promotion of health, and had a desire for holistic treatment [5]. Clearly, given the high prevalence of CAM use in the United States, the increasing integration of alternative and conventional medicine in health care settings, and the increasing consumer demand for insurance coverage for many CAM modalities, additional research on knowledge, motivations, and beliefs is a high priority.

Older users employ CAM to both manage existing health conditions and to promote wellness rather than use it for treatment alone. These findings reflect declines of health with aging, but also that older adults tend to be more health conscious than younger adults [38]. The effects of education are in the expected directions, with the most educated more likely to report wellness or combined use versus treatment alone. These findings are probably also due to the differences in beliefs according to education level, as described above [5, 37].

### Enabling resources

We find little support for net effects of enabling resources to distinguish reasons for use among CAM users. Other research documents that when comparing users to non-users, those with higher income and, in general, greater access to conventional medicine, are more likely to be users of CAM [2, 25–27]. And, a recent study by Davis and colleagues [17] that focused on just CAM users, found that wellness users had greater access to and use of conventional medical care and concluded that these individuals are high users of all types of care. Thus, it may be that our operationalization of enabling resources did not capture all of the necessary dimensions of the domain to differentiate reasons for use among CAM users. It may also be useful to ask individuals directly if they used CAM instead of conventional care, and if so, to elaborate on the specific details.

### Need

As expected, users with poorer perceived or evaluated health are much less likely to report use for wellness alone when compared to treatment alone. Contrary to our expectations, those with poorer health are not more likely to use for both wellness and treatment versus treatment alone, net of other factors. However, the bivariate analysis revealed that higher percentages of those with poorer perceived or evaluated health reported using CAM for either combined user or for treatment alone. It is possible that in the multivariate model, part of the age effects described above are capturing this effect in the combined use group. Also, our model only captures clinically diagnosed health conditions and older persons may have several other health problems for which they use CAM for combined wellness and treatment. In other studies, use of CAM among those with chronic conditions is viewed as a form of self-management and a means to improve quality of life with these conditions [5, 7, 8]. Thus, our findings provide indirect support that those with health conditions are using CAM to improve wellness even when faced with health problems.

### Personal health practices

As expected, those users engaged in a greater number of healthy behaviors are more likely to be wellness and combined users versus use for treatment. Earlier studies have found healthy behaviors such as not smoking, drinking alcohol little or in moderation, having a normal BMI, and engaging in leisure-time physical activities are more common among CAM users versus non-users [2, 4, 22, 25, 39]. A more recent study [17] found that those engaging in these healthy lifestyles behaviors were more likely to be wellness users versus combined or treatment users. Our findings suggest those users engaging in multiple healthy lifestyle behaviors may be incorporating CAM practices as part of their “wellness lifestyle” [6]. These new results also shed light on the many prior studies that reported the rather contradictory findings that users of CAM versus non-users were more likely to have poorer health but also report healthier lifestyle behaviors [2–4, 24, 25, 39]. It appears that those with existing health conditions tend to use CAM for treatment or to maintain and improve quality of life living with those health conditions. On the other hand, healthier users are inclined to engage in CAM practices to maintain positive health and wellness.

## Conclusions

The results from this study show that a sociobehavioral wellness model of CAM use has utility in distinguishing reasons for use according to wellness, combined wellness and treatment, or treatment alone. Our finding that 5 out of 6 users report reason for use as wellness or wellness in combination with treatment points to the significance of CAM as part of health self-management and wellness lifestyle. Indeed, at a time of national health care reform highlighting the importance of health and wellness and employers turning to wellness to improve worker performance and well-being, these findings suggest a central role of CAM in those public health endeavors.

## Appendix

List of CAM items in NHIS, 2007

Alternative Medical Systems

1. Acupuncture
2. Ayurveda
3. Homeopathic treatment
4. Naturopathy
5. Curandero
6. Espiritista
7. Hierbero or Yerbera
8. Shaman
9. Botanica
10. Native American health or Medicine Man
11. Sobador

Biologically based therapies

12. Chelation therapy
13. Nonvitamin, nonmineral natural products
14. Vegetarian diet
15. Macrobiotic diet
16. Atkins diet
17. Pritikin diet
18. Ornish diet
19. Zone diet
20. South Beach

Manipulative and body based therapies

21. Chiropractic or osteopathic manipulation
22. Massage
23. Feldenkreis
24. Alexander technique
25. Pilates
26. Trager Psychophysical Integration

Mind-body therapies

27. Biofeedback
28. Meditation
29. Guided imagery
30. Progressive relaxation
31. Deep breathing exercises
32. Hypnosis
33. Yoga
34. Tai chi
35. Qi gong
36. Energy healing/Reiki
